# Physician-modified fenestrated/inner-branched endovascular aortic repair for distal aortic arch aneurysm after stent graft migration: a case report

**DOI:** 10.1186/s44215-026-00272-3

**Published:** 2026-09-22

**Authors:** Yutaro Hori, Tsuyoshi Shibata, Shingo Tsushima, Kenta Yoshikawa, Shun Hayasaka, Hirokazu Sugiura, Hajime Maeda, Tomohiro Nakajima, Junji Nakazawa, Ayaka Arihara, Kenichi Kato, Shigeki Komatsu, Masato Yonemori, Yutaka Iba

**Affiliations:** 1 School of Medicine, Sapporo Medical University, Sapporo, Hokkaido Japan; 2 Division of Cardiovascular Surgery, Department of Surgery, Sapporo Medical University, 291 Minami 1-Jo Nishi 16-Chome, Chuo-Ku, Sapporo-Shi, Hokkaido, 060-8543 Japan; 3 Department of Cardiovascular Surgery, Hakodate Municipal Hospital, Hakodate, Hokkaido Japan; 4 Division of Radiology and Nuclear Medicine, Sapporo Medical University Hospital, Sapporo, Hokkaido Japan; 5 Department of Physical Therapy, Sapporo City General Hospital, Sapporo, Hokkaido Japan

**Keywords:** Physician-modified endograft, Aortic arch aneurysm, Endovascular aortic repair

## Abstract

**Backgrounds:**

Open surgical repair remains the gold standard for aortic arch aneurysms, and endovascular treatment options are limited in high-risk patients. We report successful treatment of a distal aortic arch aneurysm using physician-modified fenestrated/inner-branched endovascular aortic repair (PM-F/iBEVAR), suggesting its feasibility as an alternative strategy.

**Case presentation:**

A 50-year-old man presented with a distal aortic arch aneurysm enlargement after stent graft migration following preemptive thoracic endovascular aortic repair. The patient had significant neurological sequelae with impaired activities of daily living and was considered unsuitable for conventional arch replacement after multidisciplinary discussion. PM-F/iBEVAR was performed, and an inner branch was sutured only to the left subclavian artery in this patient. Although a type II endoleak was detected on postoperative day 3, the type Ia endoleak had resolved, and patency of the arch branch vessels was well maintained. The patient was discharged without any complications. At 6 months, the aneurysm remained stable without enlargement.

**Conclusions:**

This case report suggests that PM-F/iBEVAR may be a feasible option for patients at high risk for open surgical repair with anatomically challenging distal aortic arch aneurysms.

## Background

Open surgical repair remains the standard treatment for distal aortic arch aneurysms [1]. However, treatment remains challenging in patients unsuitable for open repair. We report successful physician-modified fenestrated/inner-branched endovascular aortic repair (PM-F/iBEVAR) in such a patient.

## Case presentation

**Fig. 1:**
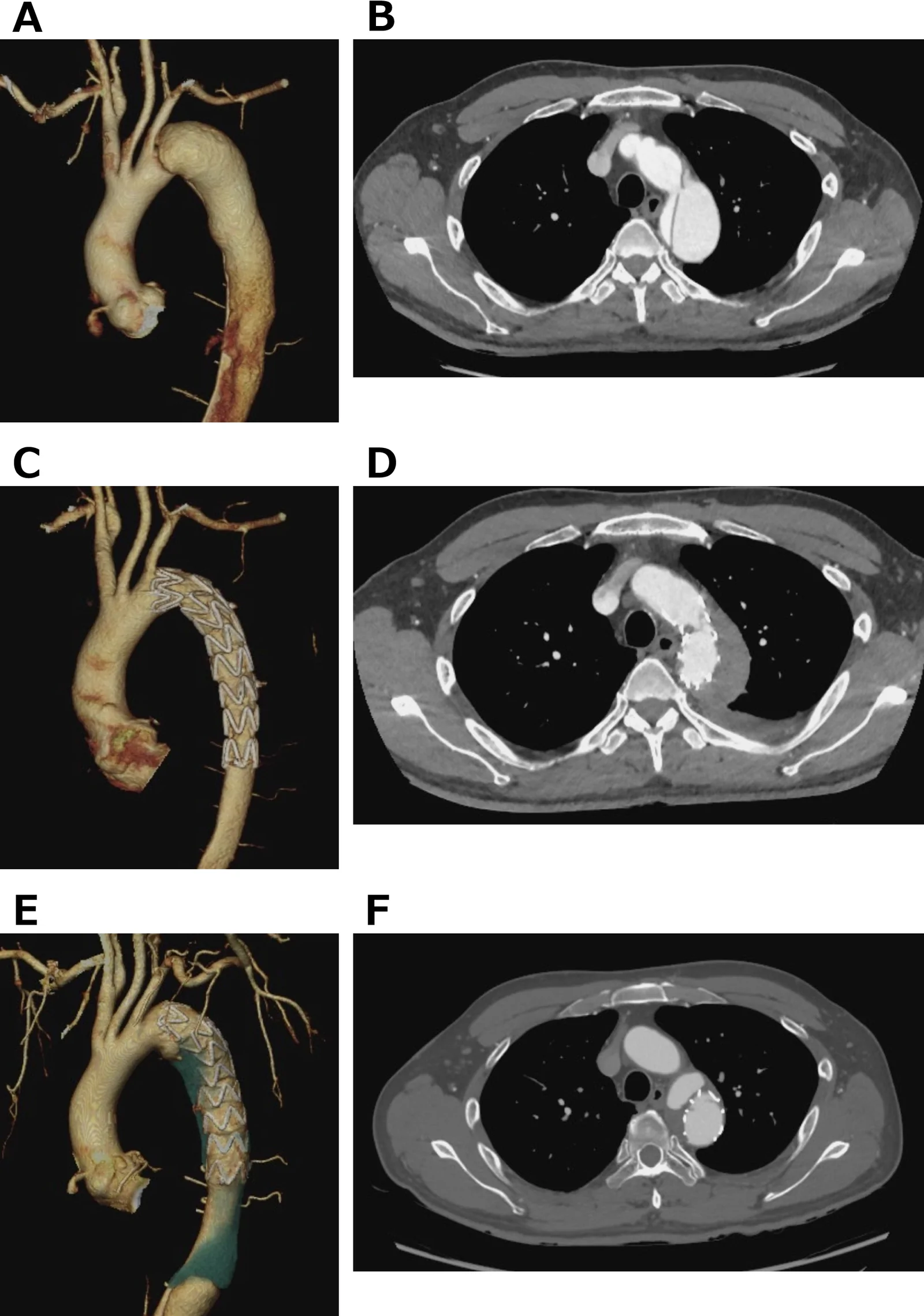
Serial CT findings during the clinical course. **A** 3D CT after the onset of acute type B aortic dissection and prior to preemptive TEVAR. **B** Axial contrast-enhanced CT after the onset of acute type B aortic dissection and prior to preemptive TEVAR. **C** Postoperative 3D CT after preemptive TEVAR. **D** Postoperative contrast-enhanced CT after preemptive TEVAR. **E** Preoperative 3D CT before PM-F/iBEVAR. **F** Preoperative contrast-enhanced CT before PM-F/iBEVAR. CT: computed tomography; 3D: 3-dimensional; TEVAR: thoracic endovascular aortic repair; PM-F/iBEVAR: physician-modified fenestrated/inner-branched endovascular aortic repair

The patient was a man in his 50 s who had developed an uncomplicated DeBakey type IIIa acute aortic dissection in year X-4, with the entry tear located in the distal aortic arch. Although he was initially managed with conservative follow-up, he sustained a cerebral infarction in year X-3, which was highly suspected to be an aortic dissection-related embolic event. Subsequent computed tomography (CT) revealed persistent false lumen patency, and the distal aortic arch was dilated to over 40 mm. Therefore, to prevent long-term progression to a thoracoabdominal aortic aneurysm, preemptive thoracic endovascular aortic repair (TEVAR) with a zone 3 landing was performed in year X-3. A 30 mm × 26 mm × 164 mm Relay Pro NBS (Terumo Aortic, Sunrise, FL, USA) stent graft was used. The proximal landing zone diameter was approximately 27 mm, and a 30-mm stent graft was selected at the previous hospital. The oversizing rate was approximately 11%. During subsequent follow-up, a CT performed in year X revealed persistent blood flow within the proximal false lumen due to a type Ia endoleak. The diameter of the distal aortic arch increased from 33 mm in year X-2 to 44 mm in year X, representing aneurysmal degeneration; therefore, the patient was referred to our institution for further intervention (Fig. 1). His medical history included multiple cerebral infarctions, intracerebral hemorrhage, and renal dysfunction, with residual right-sided limb ataxia and dysarthria. These neurological sequelae resulted in impaired activities of daily living, and the patient was considered unsuitable for conventional arch replacement after multidisciplinary discussion. Therefore, open surgical repair was deemed high risk, and PM-F/iBEVAR was planned.

**Fig. 2:**
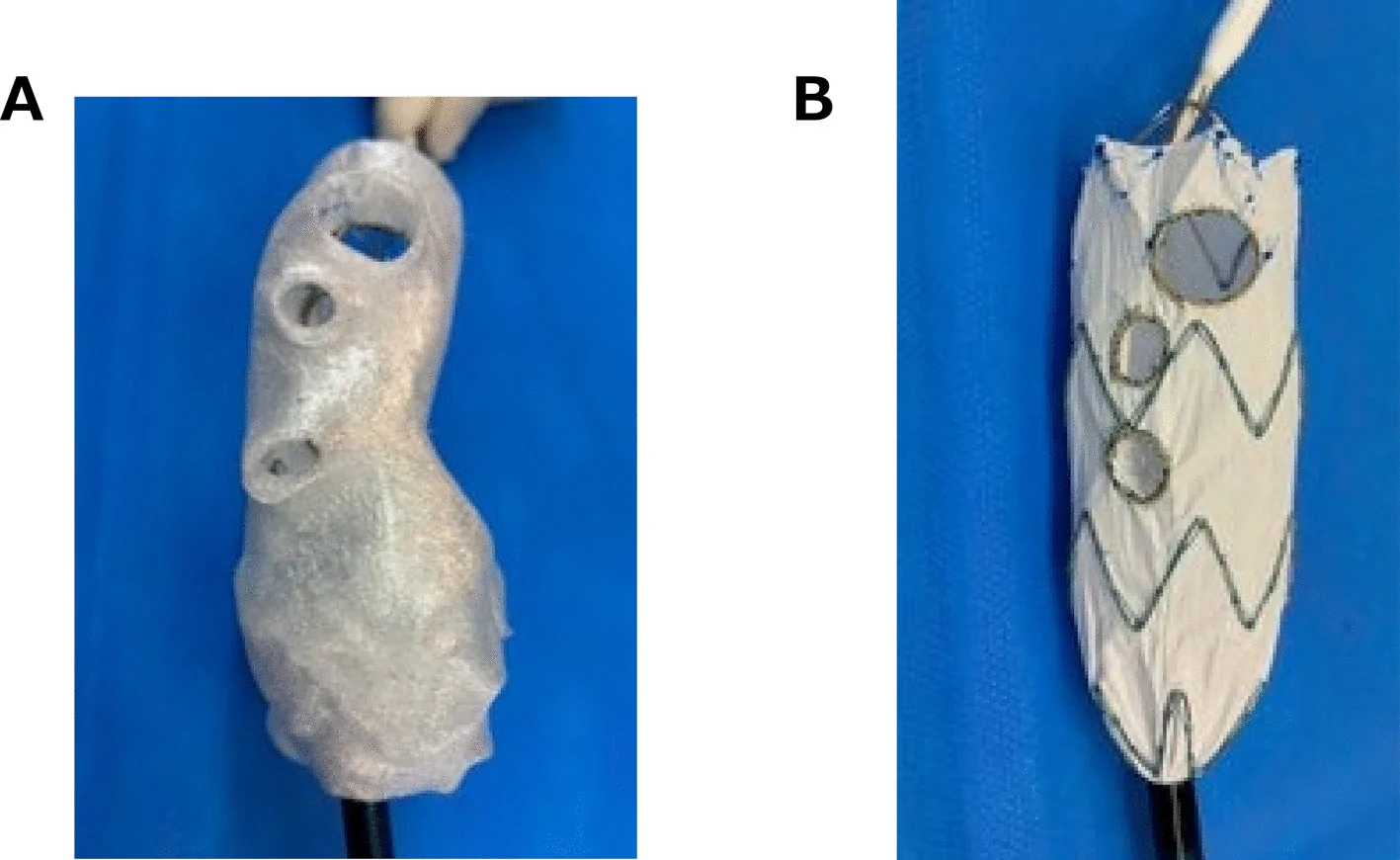
**A** Three-dimensional model. This model was created based on preoperative measurements to reproduce the patient’s aortic arch anatomy. **B** Physician-modified stent graft. Fenestrations were created for the BCA, LCA, and LSCA, and an inner branch was sutured to the fenestration corresponding to the LSCA. BCA: brachiocephalic artery; LCA: left common carotid artery; LSCA: left subclavian artery

A 34 mm × 30 mm × 161 mm Zenith Alpha Thoracic Endovascular Graft (Cook Medical, Bloomington, IN, USA) was prepared under sterile conditions in the operating room and modified by adding three fenestrations for the brachiocephalic artery (BCA), left common carotid artery (LCA), and left subclavian artery (LSCA). The Zenith Alpha was selected for this procedure because its ease of fenestration and re-sheathing makes it an exceptionally useful base device for physician-modified stent grafts [2]. The stent graft was first partially deployed from the delivery system within a sterilized three-dimensional model accurately reproducing the aortic arch. After confirming the anatomical relationship of the aortic arch branches, the polyester graft fabric was cauterized using electrocautery to create a 15 × 10 mm fenestration for the BCA and 8 × 8 mm fenestrations for the LCA and LSCA. Next, a 9-mm Gore Viabahn Endoprosthesis (W. L. Gore and Associates, Flagstaff, AZ, USA) was cut to 7 mm and fashioned as an inner branch. This was attached to the LSCA fenestration using 5–0 proline running sutures. Additionally, to facilitate intraoperative orientation, ONE Snare (Merit Medical Systems, South Jordan, UT, USA) was sutured to each fenestration as a radiographic marker. Finally, the physician-modified stent graft was manually re-sheathed. This modification took 55 min (Fig. 2).

**Fig. 3:**
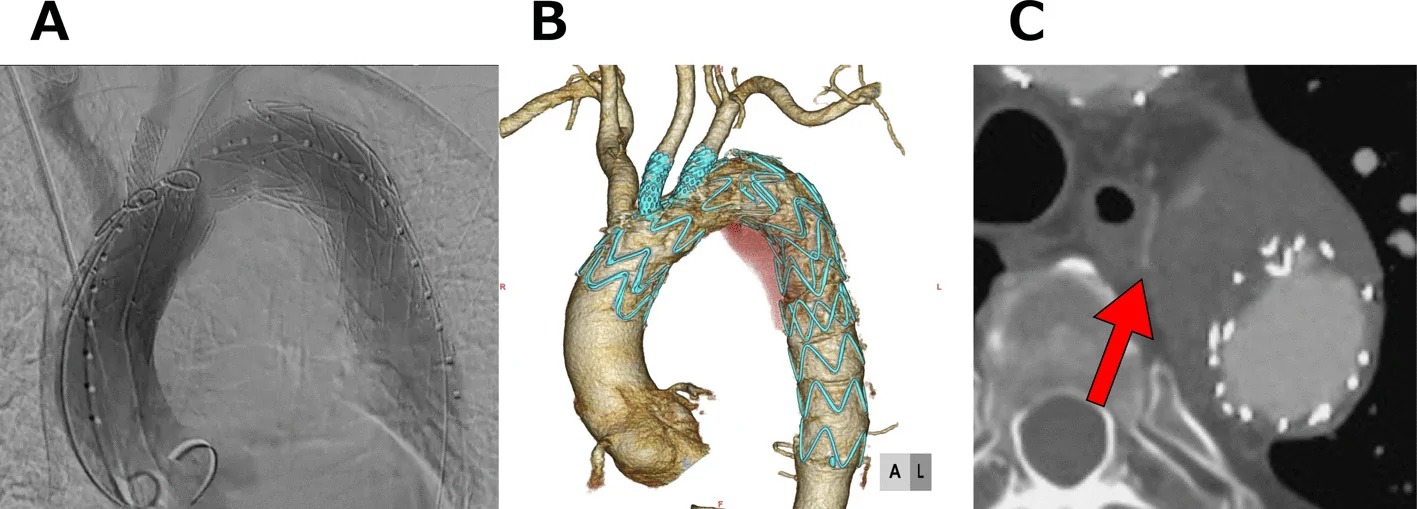
**A** Final intraoperative angiography. No obvious endoleak was observed. **B** 3D CT obtained on postoperative day 3. The type Ia endoleak had disappeared, and the patency of the aortic arch branches was good. Although a type II endoleak was observed, no findings requiring reintervention were identified. **C** Axial contrast-enhanced arterial-phase CT obtained on postoperative day 3. The bronchial arteries were enhanced as an inflow artery of a type II endoleak. The outflow arteries were not clearly visualized. 3D: 3-dimensional; CT: computed tomography

**Fig. 4:**
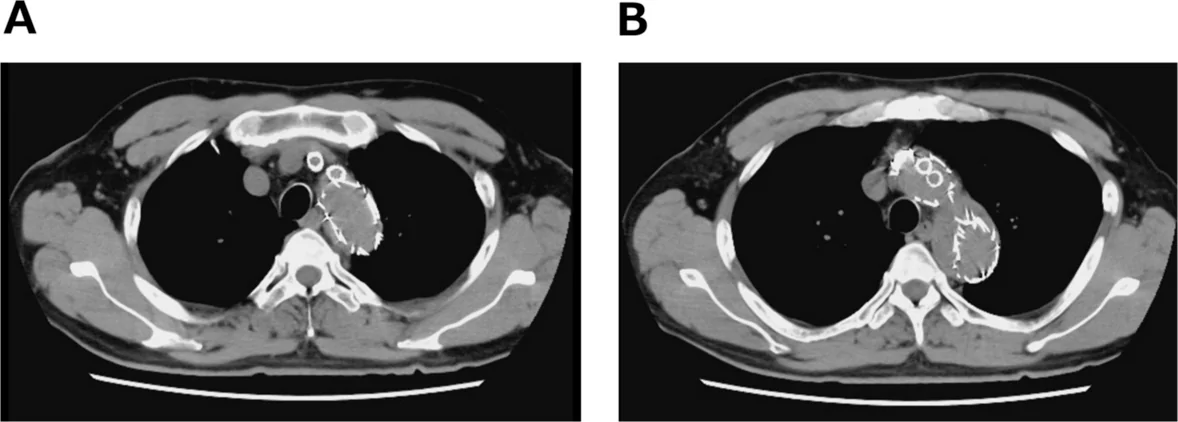
Axial plain CT images obtained at the 6-month follow-up (**A**) The supra-aortic branches remain patent. **B** The aortic aneurysm diameter measures 42 mm, representing no significant interval change. CT: computed tomography

This stent graft was advanced into the thoracic aorta via the right femoral artery. Angiography was performed to confirm the positions of the BCA, LCA, and LSCA. Based on the radiographic markers, the fenestrations were aligned with the arch branch vessels. Rapid ventricular pacing was performed at 180 bpm to ensure stability and prevent device migration during deployment. Preoperative imaging had confirmed an adequate ascending aortic length of 79 mm, which allowed to avoid over-advancing the device, thereby minimizing the risk of interference with the aortic valve. Through left cervical and upper extremity approaches, 9 × 39 mm Viabahn VBX balloon-expandable endoprostheses (W. L. Gore & Associates, Flagstaff, AZ, USA) were deployed in the LCA and LSCA, respectively. Final angiography confirmed the absence of endoleak. The operative time was 115 min, estimated blood loss was 100 mL, radiation exposure was 512 mGy, and the contrast volume used was 95 mL. Contrast-enhanced CT performed on postoperative day 3 demonstrated a type II endoleak originating from bronchial arteries; however, the type Ia endoleak had resolved, and patency of the arch branch vessels was well maintained. The patient was discharged without any complications (Fig. 3). At the 6-month follow-up, contrast-enhanced CT was omitted, leaving the presence of an endoleak unevaluated; however, the supra-aortic branches remained patent, and the aortic aneurysm diameter measured 42 mm, representing no significant interval change (Fig. 4).

## Discussion and Conclusions

**Fig. 5:**
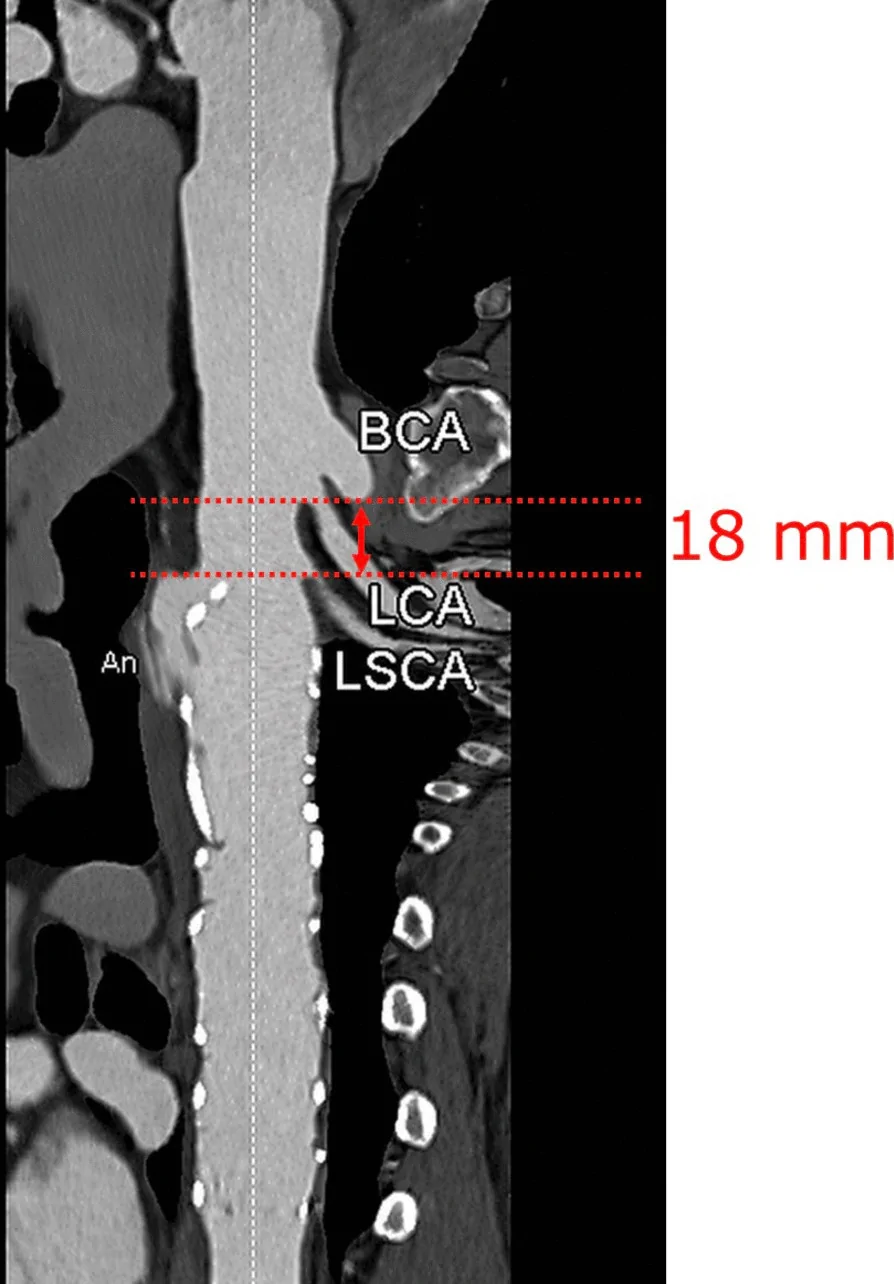
Centerline analysis. Centerline analysis revealed a proximal sealing zone length of 18 mm. An: aneurysm; BCA: brachiocephalic artery; LCA: left common carotid artery; LSCA: left subclavian artery

The predicted operative mortality rate according to the JapanSCORE was 5.3%, and the patient's pre-operative modified Rankin Scale score was grade 3 or 4. Although the predicted operative risk was not prohibitively high based on the scoring system, the patient presented with significantly impaired activities of daily living and residual neurological deficits secondary to a prior cerebral infarction and intracerebral hemorrhage. Following a multidisciplinary discussion within our aortic team, conventional open arch repair was deemed inappropriate due to these cumulative clinical vulnerabilities, and an endovascular approach was consequently selected. As an alternative, zone 2 TEVAR with either 1-debranching or LSCA fenestration was considered. Zone 2 landing was not selected because the proximal sealing zone measured only 18 mm by centerline analysis, indicating inadequate sealing (Fig. 5). Similarly, the GORE TAG Thoracic Branch Endoprosthesis (W. L. Gore and Associates, Flagstaff, AZ, USA), which is designed for zone 2 landing, was not considered suitable because it would not have provided an adequate proximal sealing zone in this patient's anatomy. In addition, 1-debranching would have required additional surgical procedure for bypass creation and carried the risk of bypass graft occlusion. When considering a zone 1 landing, secure deployment was deemed difficult due to an extremely short inter-vascular distance between the BCA and LCA, which originated from almost the same location. LSCA fenestration was also considered suboptimal because of the potential risk of endoleak at the fenestration site. For these reasons, these approaches were not chosen. Commercially available multibranched stent grafts or patient-specific custom-made stent grafts for zone 0 landing have not yet been approved for clinical use in Japan. Retrograde in situ branched stent-grafting (RIBS) and in situ fenestration (ISF) offer notable advantages, including eliminating the need for pre-procedual modification and immediate readiness for emergencies. However, concerns remain regarding the risk of procedural cerebral embolism and the risk of type IIIc endoleaks. There is also a risk of damaging the vascular wall or the graft material itself during the fenestration process [3]. In the present case, the acute angulation at the origin of the LSCA was considered a significant technical challenge for the successful application of either RIBS or ISF. Najuta Thoracic Stent Graft System (SB-Kawasumi Laboratories, Inc., Kawasaki, Kanagawa, Japan) was also considered but rejected because the steep arch anatomy increased the risk of bird-beak formation and type Ia endoleak [4, 5]. Previous studies have shown that zone 0 landing provides better proximal sealing and reduces bird-beak formation compared with zone 1 or 2 landing [4, 5]. Therefore, zone 0 PM-F/iBEVAR was selected.

Deployment of a stent graft in Zone 0 for patients with a history of aortic dissection carries a risk of retrograde type A aortic dissection (RTAD), particularly with proximal bare-stent (PBS) devices [6] To mitigate this risk, oversizing must be minimized; here, a 34 mm device was chosen for a proximal aortic diameter of 32 mm and ballooning was restricted to the absolute minimum to prevent aortic wall injury. Stent graft migration remains one of the major challenges in endovascular treatment. In this case, a NBS device had been used for the preemptive TEVAR, and subsequent migration requiring secondary intervention occurred. The use of a PBS has been suggested to reduce the risk of migration. Previous studies have shown that non-bare stent devices and steep aortic arch anatomy are associated with bird-beak formation and subsequent stent graft migration [7, 8]. These factors likely contributed to migration in the present case.

Preventing fenestration-related endoleaks is crucial in PM-F/iBEVAR. Kurimoto et al. reported that fenestration-related endoleaks requiring reintervention are relatively common after zone 0fenestrated TEVAR [9]. In the present case, an inner branch was created only for the LSCA because it was closest to the aneurysm and considered at greatest risk of endoleak. Bridging stents were deployed in the LCA and LSCA but not in the BCA, where sufficient sealing was expected because of the long distance from the aneurysm and good graft apposition.

Previous reports of PM-F/iBEVAR for distal aortic arch aneurysms have demonstrated favorable short-term outcomes, including high branch patency, absence of endoleaks, and freedom from secondary intervention [10–12]. These findings support the feasibility of PM-F/iBEVAR in carefully selected patients.

This study has several limitations. The long-term durability and safety of PM-F/iBEVAR remain unclear, and careful follow-up is required because endoleaks may occur and require secondary intervention. Zone 0 endovascular repair may also increase the risk of RTAD [6]. Furthermore, PM-F/iBEVAR is a technically demanding off-label procedure that should be reserved for carefully selected patients treated at experienced aortic centers with appropriate institutional and ethical approval. Because contrast-enhanced CT was not performed at the 6-month follow-up, persistent or recurrent endoleak could not be definitively excluded. Although the type II endoleak observed in this case may resolve spontaneously, continued surveillance is required. PM-F/iBEVAR was successfully used as a rescue strategy after TEVAR failure caused by stent graft migration in a patient with a distal aortic arch aneurysm requiring branch reconstruction.

## Data Availability

Not applicable.

## Abbreviations

PM-F/iBEVAR: Physician-modified fenestrated/inner-branched endovascular aortic repair,
CT: Computed tomography,
TEVAR: Thoracic endovascular aortic repair,
BCA: Brachiocephalic artery,
LCA: Left common carotid artery,
LSCA: Left subclavian artery,
RIBS: Retrograde in situ branched stent-grafting
ISF: In situ fenestration
RTAD: Retrograde type A aortic dissection
PBS: Proximal bare-stent
NBS: Non-bare stent

## References

[1] Ogino H, Iida O, Akutsu K, et al. JCS/JSCVS/JATS/JSVS 2020 guideline on diagnosis and treatment of aortic aneurysm and aortic dissection. Circ J. 2023;87:1410–621. 37661428 10.1253/circj.CJ-22-0794

[2] Chait J, Tenorio ER, Mendes BC, et al. Impact of gap distance between fenestration and aortic wall on target artery instability following fenestrated-branched endovascular aortic repair. J Vasc Surg. 2022;76:79-87.e4. 35181519 10.1016/j.jvs.2022.01.135

[3] Ohki T, Maeda K, Baba T, et al. Early clinical outcomes of retrograde in situ branched stent grafting for complex aortic arch aneurysms. J Vasc Surg. 2022;75:803-811.e2. 34742885 10.1016/j.jvs.2021.10.031

[4] Sato H, Fukada J, Tamiya Y, et al. Long-term clinical outcomes of thoracic endovascular aortic repair for arch aneurysms with the Najuta thoracic stent-graft system. Ann Vasc Dis. 2020;13:384–9. 33391555 10.3400/avd.oa.20-00102 PMC7758573

[5] Kudo T, Kuratani T, Shimamura K, et al. Determining the optimal proximal landing zone for TEVAR in the aortic arch: comparing the occurrence of the bird-beak phenomenon in zone 0 vs zones 1 and 2. J Endovasc Ther. 2020;27:368–76. 32242769 10.1177/1526602820914269

[6] Chen Y, Zhang S, Liu L, et al. Retrograde type A aortic dissection after thoracic endovascular aortic repair: a systematic review and meta-analysis. J Am Heart Assoc. 2017;6:e004649. 28939705 10.1161/JAHA.116.004649 PMC5634245

[7] Banno H, Akita N, Fujii T, et al. Proximal bare stent may reduce bird-beak configuration, which is associated with distal migration of stent graft in the aortic arch. Ann Vasc Surg. 2019;56:108–13. 30342207 10.1016/j.avsg.2018.08.081

[8] Marrocco-Trischitta MM, de Beaufort HW, Secchi F, et al. A geometric reappraisal of proximal landing zones for thoracic endovascular aortic repair according to aortic arch types. J Vasc Surg. 2017;65:1584–90. 28222992 10.1016/j.jvs.2016.10.113

[9] Kurimoto Y, Maruyama R, Ujihira K, et al. Thoracic Endovascular Aortic Repair for Challenging Aortic Arch Diseases Using Fenestrated Stent Grafts From Zone 0. Ann Thorac Surg. 2015;100:24–32. 25986100 10.1016/j.athoracsur.2015.01.071

[10] Shibata T, Iba Y, Yasuhara K, et al. Multicentre retrospective analysis of physician-modified fenestrated/inner-branched endovascular repair for complex aortic aneurysms. Eur J Cardiothorac Surg. 2024;66:ezae404. 39531264 10.1093/ejcts/ezae404

[11] Tsushima S, Shibata T, Iba Y, et al. Initial Outcomes of Physician-Modified Inner-Branched Endovascular Repair for Distal Aortic Arch Aneurysm. J Clin Med. 2024;14:39. 39797122 10.3390/jcm14010039 PMC11722400

[12] Zhang Y, Shen J, Yang P, et al. Physician-modified endograft with triple inner branches for extensive aortic arch aneurysm. J Endovasc Ther. 2022;29:623–6. 34839726 10.1177/15266028211059439

